# Secreted frizzled-related protein 3 was genetically and functionally associated with developmental dysplasia of the hip

**DOI:** 10.18632/aging.202815

**Published:** 2021-04-04

**Authors:** Renjie Xu, Fei Zhang, Junlan Lu, Kexin Wang, Peng Pan, Ye Sun, Yuxin Zhang

**Affiliations:** 1 Department of Rehabilitation Medicine, Kunshan Rehabilitation Hospital, Suzhou 215300, Jiangsu, People’s Republic of China; 2 Department of Orthopaedics, Huai’An People’s Hospital Of Hongze, Hongze 223100, Jiangsu Province, People’s Republic of China; 3 School of Kinesiology, Shanghai University of Sport, Yangpu 200438, Shanghai, People’s Republic of China; 4 Department of Anesthesiology, KunShan Hospital of Traditional Chinese Medicine, Kunshan 215300, Jiangsu, People’s Republic of China; 5 Department of Orthopaedics, The First Affiliated Hospital of Nanjing Medical University, Nanjing 210029, Jiangsu, People’s Republic of China; 6 Department of Rehabilitation Medicine, Shanghai Ninth People’s Hospital Affiliated to Shanghai Jiao Tong University School of Medicine, Huangpu 200011, Shanghai, People’s Republic of China

**Keywords:** developmental dysplasia of the hip, SNP, cell adhesion, chondrogenesis, miRNA

## Abstract

Background: Developmental dysplasia of the hip (DDH) is the most common joint disease in child orthopedics. Secreted Frizzled-Related Protein 3 (FRZB) plays an important role in joint development. however, no direct association between *FRZB* and DDH has been demonstrated.

Methods: Analysis of genotype distribution and allele frequency for detected single nucleotide polymorphisms (SNP) of *FRZB* was performed. *FRZB* expression was assayed in DDH joint tissues. Further experiments to identify the chondrogenic properties of *FRZB* were conducted. Potential upstream miRNAs for *FRZB* were assayed in DDH.

Results: Significant difference in genotype distribution for rs3768842 (OR=1.46, P=0.0081) and rs2242040 (OR=0.65, P=0.0067) was found. DDH joint tissues showed significantly higher *FRZB* expression. *FRZB* demonstrated chondrogenic and anti-hypertrophic properties *in vitro*. *FRZB* modulated cell adhesion pathway and cell spreading by regulating integrins expressions. Upstream miRNAs regulating FRZB expression were identified in DDH synovial fluid. Experiments indicated that downregulated miRNA-454 caused FRZB upregulation in DDH joint.

Conclusion: Dysregulated *FRZB* and its loci were associated with DDH. As a Wnt antagonist with chondrogenic properties, *FRZB* modulated cell adhesion pathway and cell spreading by regulating integrins expressions. FRZB in multiple DDH joint tissues might be mediated by the dysregulated miRNA expression profiles in the joint synovial fluid.

## INTRODUCTION

Developmental dysplasia of the hip (DDH) is a common hip joint disease in child orthopedics. The incidence of DDH is approximately 1 in 1000 live births, with a higher ratio in women [1, 2]. DDH broadly encompasses a spectrum of pathologic hip disorders in which hips are unstable, mainly including congenital dislocation/subdislocation of the hip and acetabular dysplasia in clinical. It is not only the concomitant physical appearance problems and activity limitation, but also the long-term complications such as hip osteoarthritis (OA) and femoral head necrosis that would severely influence the patients’ quality of life. The etiology of DDH has not been fully clarified. Multidisciplinary research indicated the environmental and genetic components of this disease. Although perinatal factors as breech presentation, high birthweight were considered to contribute to the occurrence of DDH [2], the important role of genetic factors cannot be neglected. Genes like *TBX4*, *ASPN* and *GDF5* have been reported in DDH development in multiple populations [3–6], providing a good basis for genetic research on DDH.

Frizzled-related protein (*FRZB*) is a secreted Wnt antagonist [7–9]. *FRZB* was shown indispensable in chondrogenic differentiation of stem cells and could prevent hypertrophic differentiation of articular chondrocytes [10]. Cartilage integrity loss was found in *Frzb*-knockout mice, causing increased cartilage damage with age [11]. The important role for *FRZB* in joint homeostasis was further supported by shifted extracellular matrix components and decreased chondrocyte proliferation in *Frzb*-knockout mice [11, 12]. Polymorphisms rs288326 and rs7775 in gene *FRZB* were found to be associated with hip OA [13–16]. A case-control study of 451 Caucasian women with hip OA demonstrated that rs288326 and rs7775 in *FRZB* gene were also related to the proximal femur shape, and the presence of rs288326 even mediated the association between hip shape and OA [14]. Given the significance of *FRZB* in joint development, we assumed that *FRZB* might be involved in DDH development.

In current study, we report dysregulated *FRZB* and its loci in association with DDH. Our findings suggest that FRZB, as a Wnt antagonist with chondrogenic properties, modulated cell adhesion pathway and cell spreading by regulating integrins expressions. Moreover, FRZB in multiple DDH joint tissues might be mediated by the dysregulated miRNA expression profiles in the joint synovial fluid.

## MATERIALS AND METHODS

### Patient collection and SNP genotyping in DDH patients and control

Between March 2014 and May 2016, a total of 386 patients with DDH retrieved from the department of orthopedic surgery and 558 healthy controls retrieved from physical examination department in the first affiliated hospital of Soochow University were enrolled to perform a case-control association study. All cases were confirmed to have unilateral or bilateral DDH (diagnosed according to clinical criteria and radiographic evidence). All controls had no symptom or history of DDH. Patients who had any systematic syndrome were excluded from the study. The study was approved by the ethical committee of the Soochow University, and written informed consent was obtained from the study subjects, whereby enrolled minors/children in the study would be signed by the guardians’ representative.

Genomic DNA was isolated from all the subjects either from buccal swabs with the DNA IQ System (Promega) or the peripheral blood with the NucleoSpin Kit (Macherey-Nagel GmbH and Co. KG). A case-control association study was performed by lab technician blinded to the sample status. SNPs in the current study were tag SNPs selected with the Haploview software (version 4.2). These loci were subsequently genotyped by Taqman assay both for cases and controls. Genetic analysis, data retrieval and the statistical processing are independently completed and reviewed by two persons. To ensure the accuracy of genotyping, 5% of the studied subjects were randomly selected for duplication and they had 100% concordance, indicating reliability of genotyping results.

### FRZB expression in tissue samples of DDH patients and controls

Total RNA in clinical samples was isolated using RNA purification kits (ThermoFisher Scientific). Control samples were collected from patients with femoral head fracture or destructive amputation injury, and tissue samples of the hip joint were retrieved in the hip arthroplasty surgery or amputation surgery. A total 2 mg RNA was reverse transcribed to cDNA using a cDNA Reverse Transcription Kit (Applied Biosystems). *FRZB* expression in the tissue was measured by real-time PCR with gene-specific primers as follows: forward 5’-CTCATCAAGTACCGCCACTCGTG -3’, reverse 5’- CCGGAAATAGGTCTTCTGTGTAGCTC -3’ for the *FRZB* gene, and forward 5’-GAGTC AACGGATTTGGTCGT-3’, reverse 5’-TTGATTTTGGAGGGATCTCG-3’ for the endogenous control gene *GAPDH*. Real-time PCR was done as previously reported [17].

### Immunofluorescence analysis of tissue sections

Immunofluorescence analysis of tissue sections was done as we previously described [17]. Briefly, sections were fixed in 4% PFA, washed with TBS-T, and then incubated with hyaluronidase (Sigma). After blocking with goat serum, sections were incubated with primary antibody for 2h at room temperature. PFA-embedded sections were deparaffinized and incubated in 1mM EDTA at 80° C for 15 mins for antigen retrieval. Primary antibodies for *FRZB* were diluted 1:200 and used (Abcam, Catalog No: ab273582). Immune complexes were detected with immunofluorescent secondary antibody using Goat Anti-Rabbit IgG H&L Alexa Fluor® 555 (Abcam, Catalog No. ab150078). DAPI (Beyotime biotechnology, Catalog No. C1002) was also used to detect the nucleus.

### Different treatment of cultured BMSC and ATDC5 *in vitro*

BMSCs were acquired from mouse bone marrow as previously described [18]. Briefly, marrow aspirates (10-20 mL) were harvested and then transferred to plastic tubes. Retrieved BMSCs were further expanded in α-MEM medium [18]. Medium was changed every 2-3 days and Passage 2 BMSCs were used for the later experiments [19, 20]. For exogenous *FRZB* group, 50 ng/ml *FRZB* (R&D, Catalog No: AAB51298) was supplemented for two weeks into the medium. *FRZB* antibody (200ng/ml, Abcam, Catalog No: ab273587) was added into some cultures to neutralize FRZB in the medium. *FRZB* knockdown was performed using *FRZB* siRNA. *FRZB* siRNA (customized from GenePharma) and negative control(GenePharma, Catalog No. A06001) were acquired from GenePharma (Shanghai, China). Sequences used to construct *FRZB*-siRNA were 3’-GGAGATTCTAAAGTCCTCTTTCAAGAGAAGAGGACTTTAGAATCTC C-5’. The siRNAs were cloned into pGPU6/Neo (GenePharma). After confirmation of inhibiting efficacies, selected *FRZB* siRNA (150nM) or negative control was used to transfect primary ATDC5 using Lipofectamine 2000 (Invitrogen, Carlsbad, CA, USA). Isolated mRNAs were used to construct raw RNA sequence data obtained using an Illumina HiSeq™2000 machine [21]. The trimmed reads of mRNA profiles were further mapped with the Novoalign software (v2.07.11). mRNA expression profiles for the *FRZB* siRNA group and control group were profiled. Difference between FRZB siRNA and control group was analyzed using a paired two-sided test and the Bonferonni test. Fold changes and p-values were calculated for each mRNA profiled. Differentially enriched mRNAs were filtered with a fold change ≥ 2.0 and a false discovery rate (FDR) < 0.05 [21]. To predict the target pathways of differentially expressed mRNAs, Gene ontology (GO) categories of the predicted genes were analyzed using DAVID Bioinformatics Database, and pathway analysis (KEGG, Wiki, Reactome, Biocarta) was performed using the EnrichR web platform [21]. Validation of protein expressions were conducted with western blot for cells with different treatments as previously described. Anti-*SOX9* antibody (Abcam, Catalog No: ab185966), Anti-*ACAN* antibody (Invitrogen, Catalog No: PA1-1746), Anti-*ITGA8* antibody (Abcam, Catalog No: ab243027), Anti-*ITGAV* antibody (Abcam, Catalog No: ab179475) were used at the concentration of 1:200 dilution. Horseradish peroxidase (Jackson ImmunoLab) was used as the secondary antibody. *GAPDH* blots were used as control with *GAPDH* antibody (1:5000 diluted, Sigma).

Immunofluorescent analysis of *ACAN* (Invitrogen, Catalog No: PA1-1746) was performed to study the generated chondrocytes in different treatment. Images were observed with a confocal microscope (Leica, Japan). Expression of *SOX9, Col2A1*, *GDF5* and *Col10A1* after 2-week culture was quantified with RT-PCR. Chondrogenic tissues formed under different treatment was stained with alcian blue staining to detect proteoglycan production. Staining mages were taken with a light microscope (Leica Microsystems, Germany).

The expression of Wnt pathway genes(*β-catenin, WNT3A* and *WNT8A*), cartilage anabolic and catabolic markers (*SOX9*, *Col1A1*, *Col2A1*, *MMP9*, *MMP13*, *ADAMTS5*) and osteogenesis markers (*Col10a1*, *RUNX2* and *OCN*) after 2-week culture was quantified and compared with RT-PCR. Primer sequences for genes in PCR assay were listed in Supplementary Table 1. Chondrogenesis was determined with alcian blue staining. GAG was quantitatively analyzed (6 vs 6) with normalization to DNA content. GAG content was compared among different treatment groups

### microRNA expression and cell transfections

Total RNA was extracted from joint synovial fluid from DDH patients and control using Trizol reagent and then qRT-PCR was conducted with a SYBR Green qPCR Kit and the ABI Detection System. The expressions of miR-454, miR301a, miR301b, miR130a and miR130b were normalized to U6 applying the 2^-ΔΔCt^ method. The primer details are listed in the supplementary file. MiRNA negative control (mimic control), miR-454 mimics or inhibitors were acquired from GenePharma. Transfection with these reagents was performed using Lipofectamine 3000 according to manufacturer’s protocol.

### Statistics

SAS software (version 9.2) was applied to analyze the association between the SNPs and DDH occurrence. Odd ratios (ORs) and 95% confidence interval (CI) were reported. Two-sided chi-squared test was used to confirm the significance of differences in allelic distribution and P<0.05 was considered significantly different. Hardy-Weinberg equilibrium (HWE) was checked with chi-squared test in DDH patients and the control.

## RESULTS

### Association between *FRZB* SNPs and the occurrence of DDH

Distribution of loci genotypes in both cases and controls conformed with HWE (p>0.05). Polymorphisms rs3768842 and rs2242070 were selected by Haploview software. Different allele frequency was noted between DDH patients and controls (p<0.05). Detailed distribution of genotype and alleles in DDH patients and healthy control has been listed (Tables 1, 2). An allele of rs3768842 was observed to have a positive association with the risk of DDH (OR=1.29, 95% CI: 1.07-1.56, p=0.0076), while the A allele of rs2242070 was found to have a negative association with the risk of DDH (OR=0.75, 95% CI: 0.62-0.90, p=0.0022). Significant difference was identified for genotype distribution (AA vs AG+GG) for rs3768842 (OR=1.46, 95% CI: 1.10-1.93, p=0.0081). For rs2242070, genotype distribution (AA vs AG+GG) also demonstrated significant difference (OR=0.65, 95% CI: 0.48-0.89, p=0.0067).

**Table 1:** Association between rs3768842 and DDH in different genetic models.

	**Genotype**				**Allele frequency**			**OR (95%CI)**	**P value**
	AA	AG	GG	No.	A	G	A vs G	1.29 (1.07-1.55)	**0.0076**
						
**Case**	136	189	61	386	0.60	0.40	AA vs others	1.46 (1.10-1.93)	**0.0081**
						
**Control**	151	293	112	556	0.54	0.46	others vs GG	1.34 (0.95-1.89)	0.090
						

OR = odds ratio, CI = confidence interval.

**Table 2:** Association between rs2242070 and DDH in different genetic models.

	**Genotype**				**Allele frequency**			**OR (95%CI)**	**P value**
	AA	AG	GG	No.	A	G	A vs G	0.75 (0.62-0.90)	**0.0022**
						
**Case**	79	202	104	386	0.47	0.53	AA vs others	0.65 (0.48-0.89)	**0.0067**
						
**Control**	158	286	114	558	0.54	0.46	others vs GG	0.69 (0.51-0.94)	**0.0184**
						

OR = odds ratio, CI = confidence interval.

### Differential *FRZB* expression was demonstrated in DDH joint tissues

**Figure 1:**
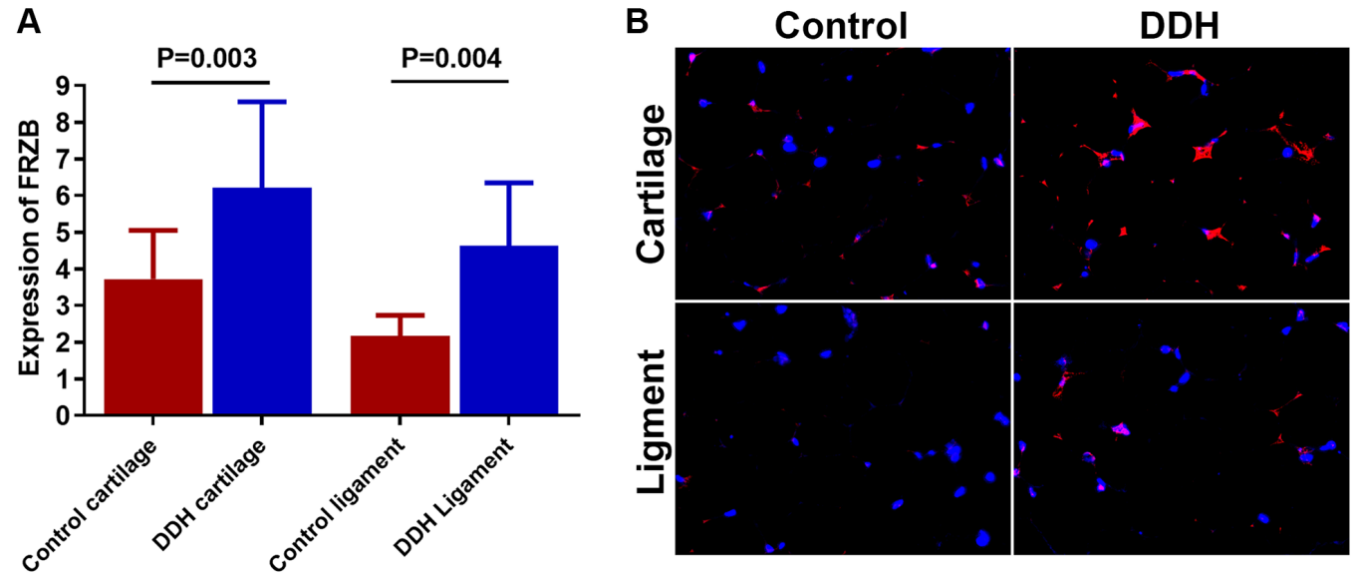
**Tissue expression of *FRZB* in patients and controls.** (**A**) DDH patients were found to have significantly higher expression of the *FRZB* in the articular cartilage and ligament as compared with the controls (2.43 ± 1.65 vs 4.05 ± 1.89, p = 0.002 for articular cartilage; 2.46 ±1.68 vs. 3.85 ± 2.73, p = 0.007 for joint ligament). (**B**) Immunofluorescent assay of *FRZB* (red) expression and nucleus (blue) in the cartilage and ligament tissues in different groups of patients observed under confocal microscopy.

DDH patients showed higher expression of *FRZB* (Figure 1A) in joint tissue samples compared to the control. (3.72 ± 1.33 vs 6.21 ± 2.35, p = 0.003 for articular cartilage; 2.18 ±0.55 vs. 4.62 ± 1.73, p = 0.004 for joint ligament). Meanwhile, immunofluorescent analysis also showed greater *FRZB* expression in the DDH tissues. (Figure 1B)

### *FRZB* induced chondrogenesis and Wnt signaling pathway *in vitro* in BMSCs

**Figure 2:**
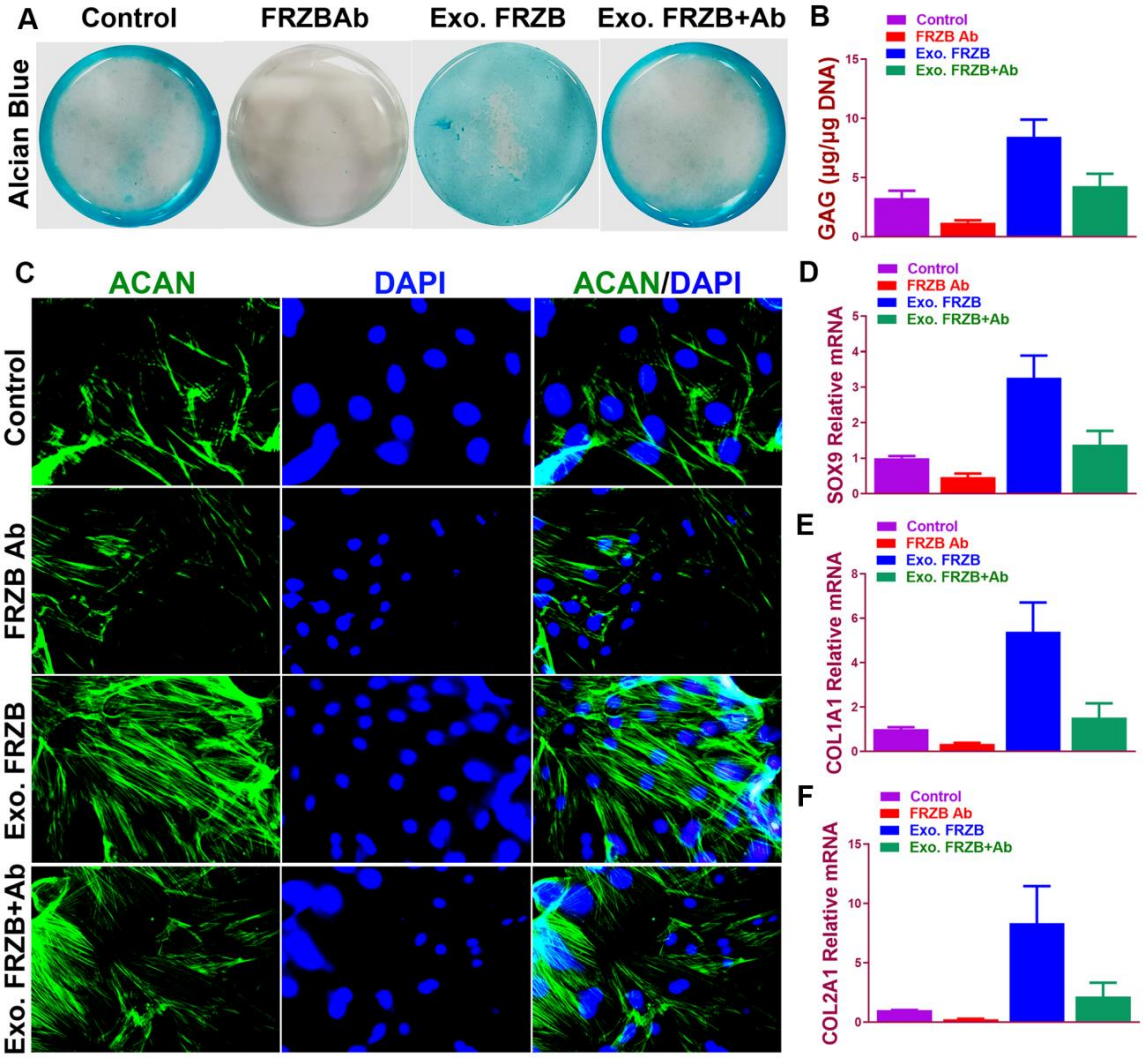
**Chondrogenic effects of *FRZB* on BMSCs *in vitro*.** (**A**) Alcian blue staining of BMSCs in different treatment groups at 2 weeks to indicate GAG production in the culture plate. (**B**) Quantification of GAG production in generated cartilaginous tissues (n=6 for each). (**C**) Immunofluorescent assay of *Aggrecan* (green) expression and nucleus (blue) in different treatment groups observed under confocal microscopy. (**D**–**F**) Expression level of chondrogenic markers for BMSCs in different treatment groups (n=6 for each) in (**A**) (n=6 for each). *P < 0.05 between control group and other groups. Data are presented as averages ± SD. One-way analysis of variance (ANOVA) with post-hoc Tukey’s B test was applied. Ab, antibody; Exo, exogenous; *ACAN*, aggrecan; GAG, glycosaminoglycan.

**Figure 3:**
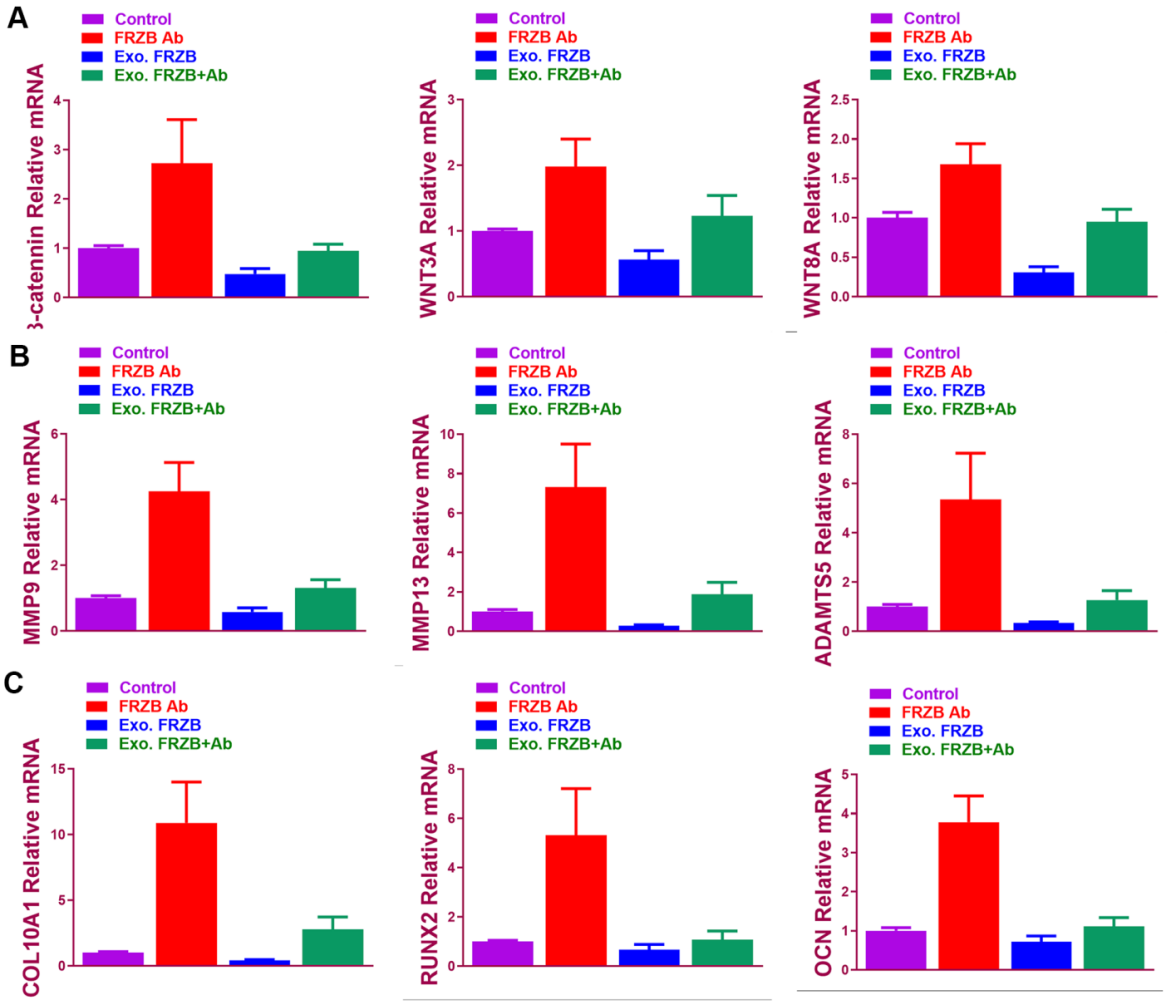
***FRZB* blocked Wnt signaling and inhibited the catabolic activities and hypertrophy in the induced chondrogenic tissues.** (**A**) Expression level of Wnt pathway markers (*β-catenin*, *WNT3A* and *WNT8A*) for BMSCs in different treatment groups (n=6 for each) (**B**) Expression level of catabolic markers (*MMP9*, *MMP13* and *ADAMTS5*) in different treatment groups (n=6 for each). (**C**) Expression level of hypertrophy and osteogenesis markers (*COL10A1*, *RUNX2* and *OCN*) for BMSCs in different treatment groups (n=6 for each). *P < 0.05 between control group and other groups. Data are presented as averages ± SD. One-way analysis of variance (ANOVA) with post-hoc Tukey’s B test was applied.

Effects of *FRZB* on chondrogenesis of BMSCs were examined *in vitro*. (Figure 2) exogenous recombinant *FRZB* enhanced GAG synthesis BMSCs, while medium supplemented with *FRZB* antibody decreased GAG deposition (Figure 2A). *FRZB* induced greater quantity of GAG deposition with higher expression of chondrogenic gene markers in the generated chondrogenic tissue (Figure 2B–2F). As a Wnt antagonist, *FRZB* blocked Wnt signaling by downregulating the expression of specific Wnt signaling genes (*β-catenin*, *WNT3A* and *WNT8A*). (Figure 3) Moreover, catabolic activities and osteogenesis of BMSCs were inhibited during the chondrogenic process induced by *FRZB*, indicative of non-hypertrophic chondrocytes induced by exogenous *FRZB* treatment (Figure 3).

### Gene expression profiles in *FRZB*-knockdown ATDC5 cells

**Figure 4:**
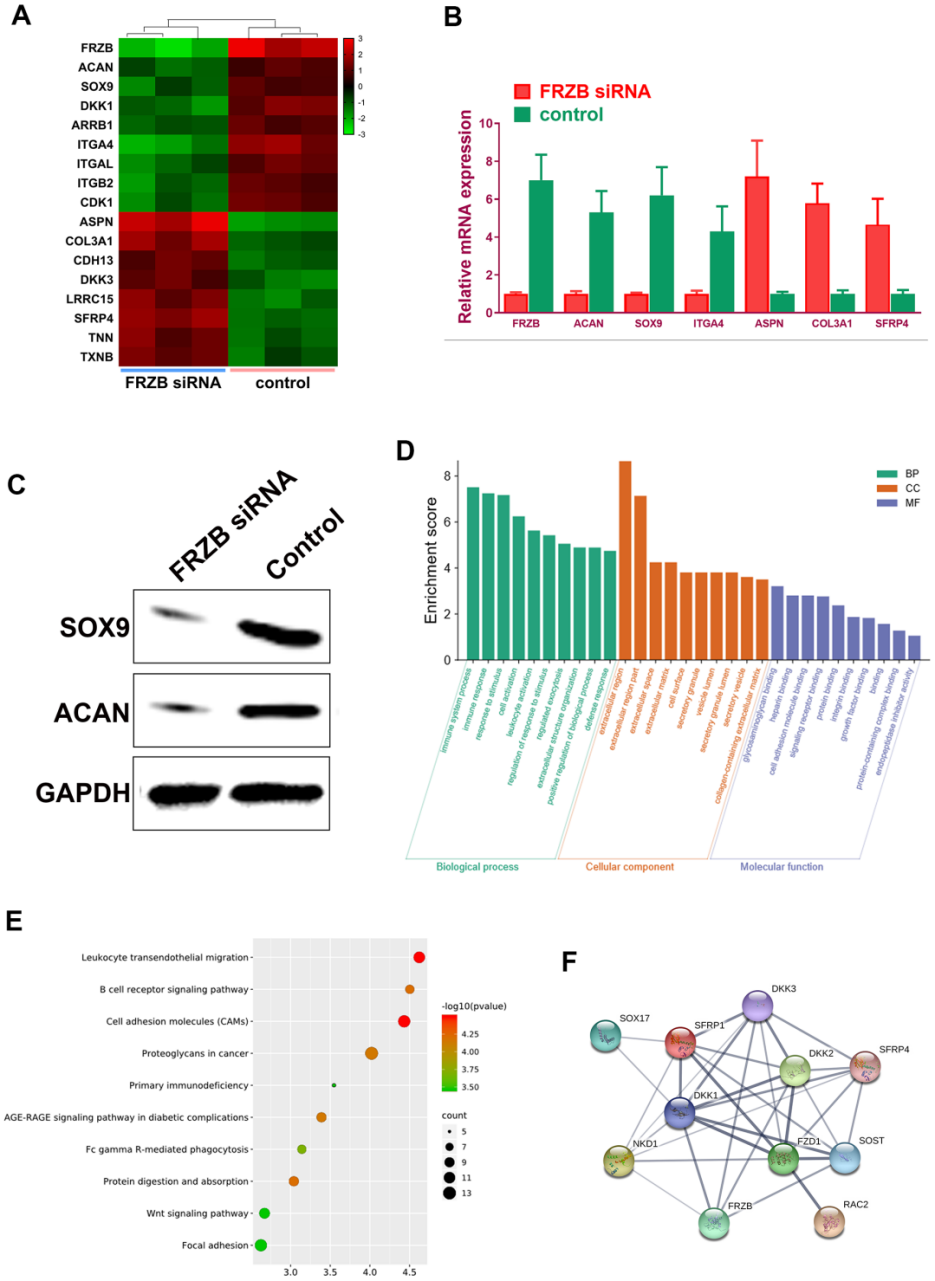
**Specific mRNA microarray profiles in *FRZB*-knockdown ATDC5 cells.** (**A**) The unique enrichment of a set of mRNA microarray profiles in *FRZB*-knockdown ATDC5 cells compared with control determined by high-throughput sequencing. Differentially enriched mRNAs were filtered with a log2(fold change) ≥ 2.0 and a false discovery rate (FDR) < 0.05. In detail, *ASPN*, *COL3A1*, *CDH13*, *DKK3*, *LRRC15* and *SFRP4* were significantly up-regulated in the *FRZB*-siRNA group, while *ACAN*, *SOX9*, *DKK1*, *ARRB1*, *ITGAL* and *CDK1* were down-regulated compared with the control group. (**B**) The enrichments of specific gene expression with *FRZB*-knockdown were validated by qPCR. * P<0.05 *vs.* control. (**C**, **D**) Western blot experiment validated decreased chondrogenesis in *FRZB*-knockdown ATDC5 cells. (**D**, **E**) GO categories and KEGG pathway analysis were conducted based on the differentially enriched mRNAs in *FRZB* knockdown cells. The enriched mRNAs in *FRZB*-knockdown ATDC5 cells were involved in a broad range of biological functions, such as response to stimulus, cell activation, extracellular matrix, cell adhesion molecular binding, signaling receptor binding and integrin binding. KEGG Pathway analysis results were closely associated with cell adhesion molecules, AGE-RAGE signaling pathway, Wnt signaling pathway and focal adhesion. (**F**) Protein-protein interaction with enriched Wnt signaling pathway-related genes. Abbreviation: GO, gene ontology.

*FRZB*-knockdown was conducted with *FRZB* siRNA in chondrogenic progenitor ATDC5 cells. Enrichment of a set of mRNA microarray profiles in *FRZB*-knockdown ATDC5 cells was compared with control through mRNA sequencing (Figure 4A). Enriched mRNAs were filtered with a log2(fold change) ≥ 2.0 and a false discovery rate (FDR) < 0.05. Specifically, *ASPN*, *COL3A1*, *CDH13*, *DKK3*, *LRRC15* and *SFRP4* were up-regulated in *FRZB*-siRNA group, and *ACAN*, *SOX9*, *DKK1*, *ARRB1*, *ITGAL* and *CDK1* were down-regulated compared with the control group (Figure 4A). Enrichments of the specific genes with *FRZB*-knockdown were further validated by qPCR and Western blot experiment, indicating significantly decreased chondrogenesis in *FRZB*-knockdown ATDC5 cells (Figure 4B, 4C). Gene ontology (GO) categories and KEGG analysis were performed with the differentially enriched mRNAs in *FRZB*-knockdown cells (Figure 4D, 4E). The enriched genes in *FRZB*-knockdown ATDC5 cells functioned in many biological processes such as response to stimulus, cell activation, extracellular matrix, cell adhesion molecular binding, signaling receptor binding and integrin binding. KEGG Pathway analysis results were closely associated with cellular adhesion molecules, AGE-RAGE signaling, Wnt signaling and focal adhesion. Protein-protein interaction network was demonstrated for enriched Wnt signaling pathway-related genes with *FRZB*-knockdown (Figure 4F).

### *FRZB* modulated cell adhesion pathway and cell spreading by regulating integrins expressions

**Figure 5:**
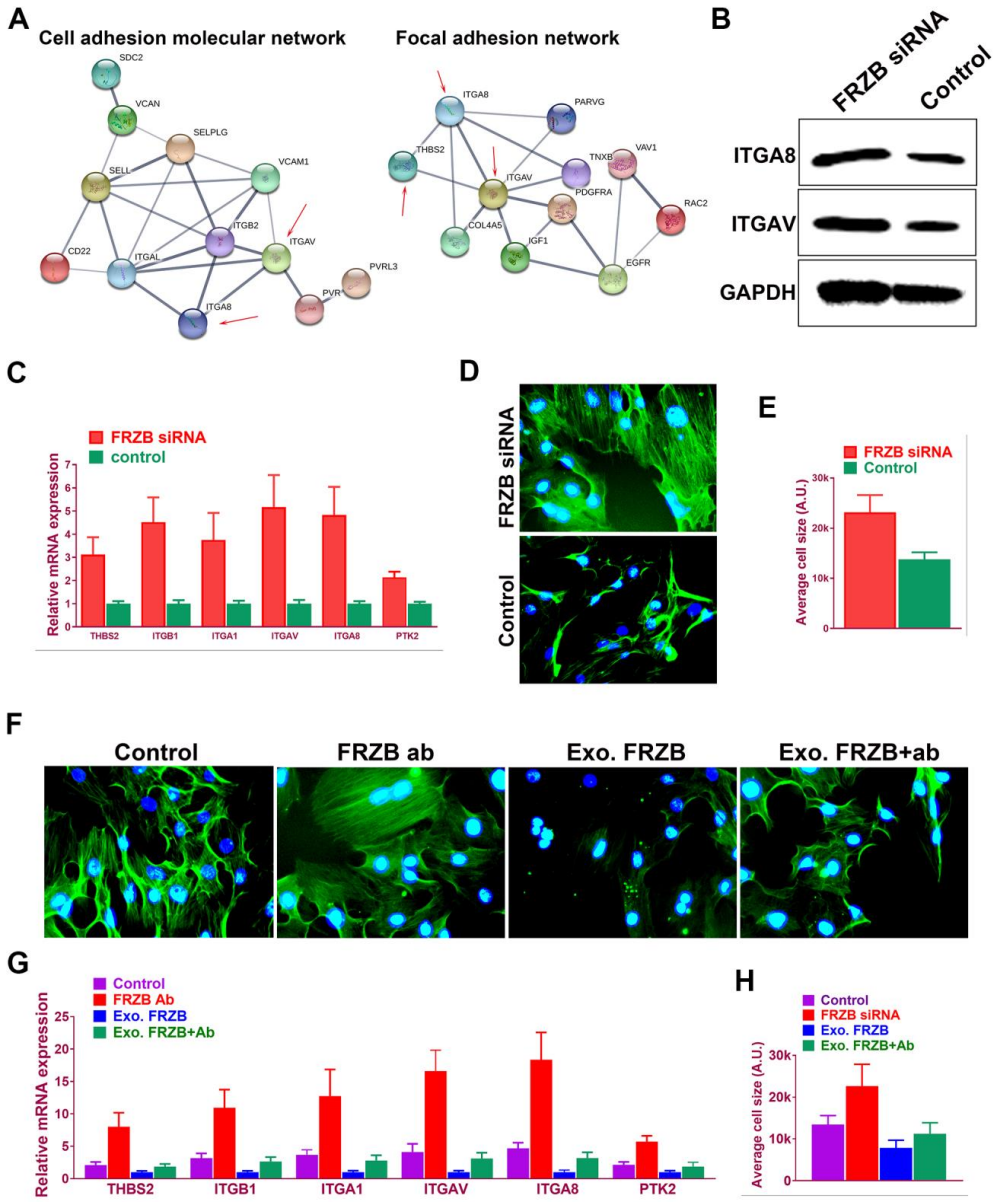
***FRZB* modulated cell adhesion pathway and cell spreading by regulating integrins expressions.** (**A**) Protein-protein interaction with enriched cell adhesion molecule and focal adhesion signaling pathway-related genes. Integrin family members *ITGA8* and *ITGAV* (red arrow) were enriched in both pathways. (**B**) Western blot experiment validated *ITGA8* and *ITGAV* expression in *FRZB*-siRNA group. (**C**) Gene expressions of cell adhesion pathway genes were also validated and quantified with RT-PCR. (**D**, **E**) Greater cell spreading was demonstrated in *FRZB*-knockdown cells with cytoskeleton staining (green: phalloidin, blue: nucleus). (**F**, **H**) Effects of exogenous *FRZB* or *FRZB* blockage on cell spreading and (**G**) its regulation of related genes in cell adhesion signaling pathway.

Microarray profiles indicated enriched cell adhesion molecules and signaling in *FRZB*-knockdown cells (Figure 5A). In previous studies, dysregulated cell adhesion signal has been reported in osteoarthritis development. Taken together, cell adhesion signaling was analyzed in *FRZB*-knockdown ATDC5. Protein-protein interaction network was derived from the gene expression with enriched cell adhesion molecule and focal adhesion signaling pathway-related genes (Figure 5A). Integrin family members *ITGA8* and *ITGAV* (red arrow) were enriched in both pathways. Western blot experiment further validated *ITGA8* and *ITGAV* expression in *FRZB*-siRNA group (Figure 5B). Gene expressions of cell adhesion pathway genes were also validated and quantified with RT-PCR, showing significantly enhanced cell adhesion signaling and increased cell size with *FRZB* knockdown (Figure 5C–5E). Greater cell spreading was demonstrated in *FRZB*-knockdown cells with cytoskeleton staining (Figure 5D, 5E). To confirm the role of *FRZB* in cell adhesion and spreading, effects of exogenous *FRZB* and *FRZB* blockage on cell spreading was conducted (Figure 5F–5H). Exogenous *FRZB* significantly inhibited cell spreading while its blockage significantly enhanced cell spreading and increased cell size (Figure 5F). Exogenous *FRZB* significantly inhibited expression of related genes in cell adhesion signaling pathway while *FRZB* blockage exerted similar effects to *FRZB* knockdown in cell adhesion signaling (Figure 5G, 5H).

### Downregulated miRNA-454 expression causes FRZB upregulation in the synovial fluid of DDH patients

**Figure 6:**
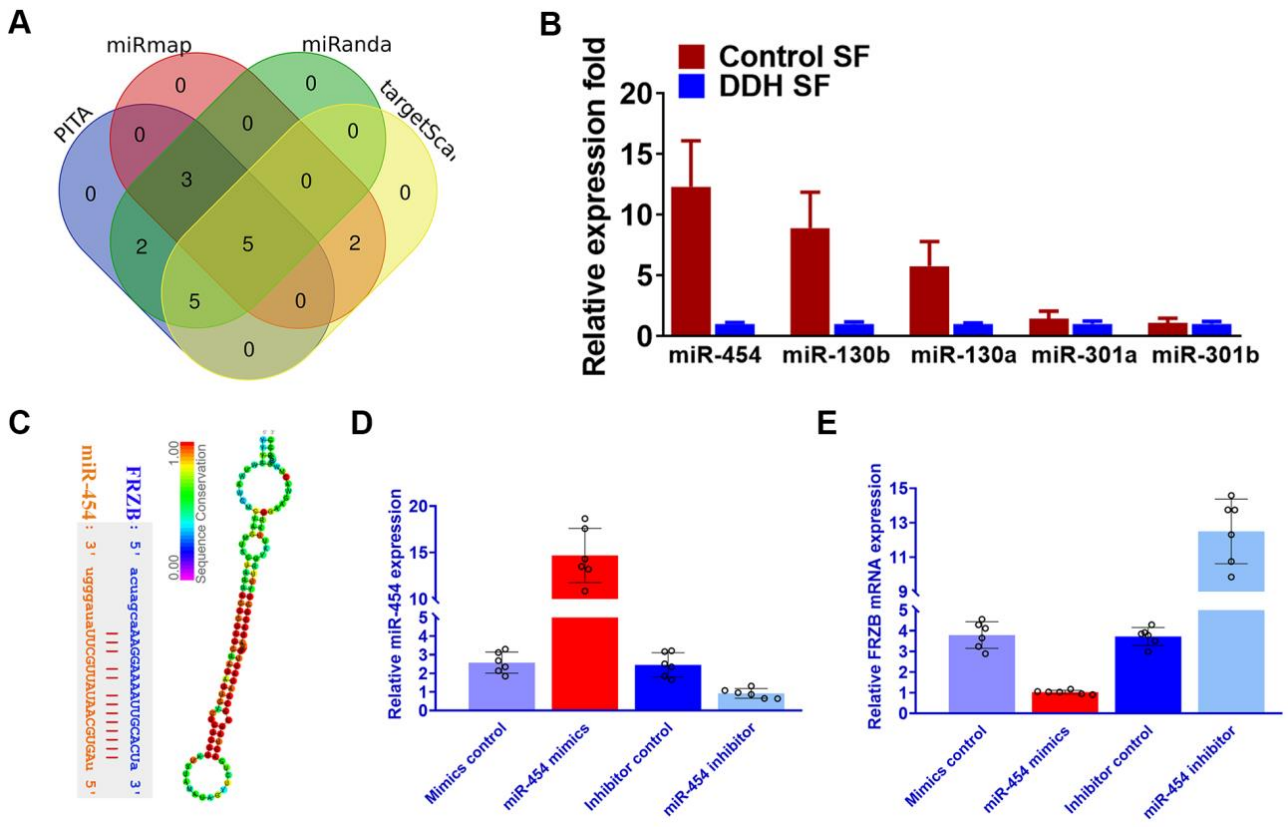
**Downregulated miRNA-454 expression causes *FRZB* upregulation in the synovial fluid of DDH patients.** (**A**) Five candidate miRNAs emerged after we queried the TargetScan, miRmap and miRanda and PITA databases. (**B**) Expression of the five microRNA candidates with qRT-PCR in synovial fluid from DDH patients compared with control samples. (**C**) Binding sites on *FRZB* for miR-454 (left panel) was predicted with targetscan and sequence alignment of miR-454 (right panel) showed high conservation among different species. (**D**) miR-454 expression by qRT-PCR in ATDC5 cells (n=6 for each) co-transfected with miR-454 mimics, miR-454 inhibitor or their negative control. (**E**) PCR results of *FRZB* expression with MiR-454 mimics and inhibitor in ATDC5.

Our results had shown that FRZB acted as a pro-chondrogenic gene in multiple DDH joint tissues. Normally, we speculate that miRNAs conveyed in the synovial fluid in the DDH samples might involve in the upregulation of FRZB in the ligament and cartilage tissues. To explore the upstream miRNAs targeting FRZB, we queried the TargetScan, miRmap and miRanda and PITA databases. Five candidates emerged after overlapping potential miRNAs from the four databases (Figure 6A). We measured the expression of the five potential microRNA candidates in synovial fluid from DDH patients and found that miR-454, miR-301a and miR-301b were significantly downregulated in DDH SF compared with normal control samples (Figure 6B). Among the three dysregulated microRNAs, miR-454 was most significantly decreased in DDH SF, so we focused on miR-454 to explore its regulation of FRZB *in vitro*. Binding sites on FRZB for miR-454 was predicted with targetscan (Figure 6C). Sequence alignment of miR-454 showed high conservation among different species. ATDC5 were transfected with miR-454 mimics, miR-454 inhibitor or their negative control. MiR-454 mimics and inhibitor successfully up or down-regulated miR-454 expression in ATDC5 (Figure 6D). Additionally, PCR results also demonstrated that overexpression with miR-454 mimics could decrease FRZB expression while blockage of miR-454 increased FRZB expression (Figure 6E), indicating that the pro-chondrogenic effects of FRZB overexpression in DDH joint tissues might be mediated by the downregulation of miR-454 in the joint SF.

## DISCUSSION

This study demonstrated significant associations between polymorphisms rs3768842 and rs2242070 in gene *FRZB* with DDH. Genotype distributions was significantly different (AA+AG vs GG) identified in cases and controls for both rs3768842 and rs2242070. DDH samples showed significantly greater *FRZB* expressions in DDH hip tissues compared to the control. *FRZB* also demonstrated chondrogenic and anti-hypertrophy properties as a wnt signaling antagonist, indicating that DDH might be attributed to the overdevelopment of femoral cartilage in patients with dysplastic hips. Potential upstream miRNAs regulating FRZB, miR-454 in specific, was identified in DDH joint synovial fluid, indicating FRZB overexpression in DDH joint tissues might be mediated by the dysregulation of miRNA expression profiles in the joint SF.

DDH is believed to be a polygenic disorder with increasing number of polymorphisms in various genes reported. Two SNPs of *CX3CR1*, rs3732378 (p=0.003) and rs3732379 (p=0.017) were identified as susceptibility loci of DDH through a case-control association study with 689 pairs of patients and controls [22]. Sun et al [23] conducted a genome-wide association study followed with a subsequent case-control study in two set of case-control groups, and identified risk allele A in rs6060373 of gene *UQCC* for DDH. However, it has to be noted that the positive findings of these genetic association studies should be interpreted with caution. A study on 310 patients with sporadic DDH and 487 controls performed by Jia et al [24] showed significant difference (p=0.001) of allele frequency in rs726252 of *PAPPA2* gene and significantly different distribution of TT genotype between cases and controls (p=0.000). While another replication study found no significant difference between 697 DDH subjects and 707 controls in neither allele frequency nor genotype distribution of rs726252 [5].

Polymorphisms in WNT-signaling genes are found to be associated with osteoarthritis, suggesting its significance in pathological musculoskeletal conditions such as DDH [25, 26]. *FRZB* has been demonstrated to be a binding-gene in WNT signaling acting as a inhibition role, the protein encoded by which is a secreted protein to regulate skeletal development [27, 28]. A number of studies have shown *FRZB* to be a determinant of hip shape formation, whereby substitutions Arg200Trp and Arg324Gly in *FRZB* sequence conferred higher risks for OA development [14, 29, 30]. Despite no direct evidence supporting the association between expression of *FRZB* and the occurrence of DDH, the close link between primary hip OA and structural hip deformities makes *FRZB* a possible susceptibility gene worthy of study [31, 32]. Polymorphisms rs2242070 in *FRZB* gene was reported to be involved in the development of osteoporosis [33, 34]. While, this is the first study reporting polymorphisms rs2242070 and rs3768842 in gene *FRZB* to be associated with DDH, which suggests a potential role of *FRZB* in the occurrence of DDH. Expression of *FRZB* was found to be lost during OA progression, which can be used as markers for staging OA at the molecular level [35]. DDH cases normally had hip joint laxity in adolescence. *FRZB* was demonstrated indispensable in chondrogenesis and could induce cartilage overdevelopment in DDH, suggesting that dysregulated chondrogenesis in DDH could be mediated by *FRZB* signaling, leading to joint cartilage overdevelopment in the dysplastic hip joints. Further therapeutic approaches might be developed in the future by targeting the FRZB-mediated signaling or inhibiting the over-chondrogenesis of the dislocated hip joint

Our data suggest that *FRZB* inhibits cell adhesion and spreading by dampening integrin signaling in chondrogenic progenitors. Integrins are the major receptor to interact with the surrounding ECM of developing chondrocytes. Integrin-ECM

Integrin-ECM was previously reported in osteogenesis and inhibition of chondrogenesis [36]. Increased FAK activation inhibits chondrogenesis, while FAK is decreased in chondrocytes [37]. Furthermore, Cell-ECM has been intensively studied osteoarthritis occurrence. Cartilage injury would induce greater catabolism by *MMP13* and *ADAMTS5*, causing further cartilage damage and releasing of ECM molecules into SF. ECM catabolite served as integrin ligands and further triggered catabolism of the articular tissues (23, 25). *FRZB* alleviates cartilage catabolism by inhibition of the activated integrins. significantly reducing cell spreading and focal adhesions. Further study is needed to address focal adhesion signaling in OA development. *FRZB* knockdown or blockage significantly reduced expression of chondrogenic markers, while exogenous *FRZB* was therapeutic and restored the chondrogenic phenotype. Integrin signal blockage has been tested for arthritis therapy (68–70). We have shown *FRZB* functions in chondrogenesis, and protects chondrocytes from destructive signals and integrin activation. *FRZB* is secreted and reduces integrin signaling by interacting with integrin. Experiments on FRZB function would shed light on new therapeutics for integrin-targeted diseases. Further replication studies on the association between *FRZB* and DDH might help early diagnosis of DDH in the future. Downregulation of miR-454 was demonstrated in DDH SF, and possibly caused the overexpression of FRZB in multiple DDH joint tissues. Dysregulation of miRNAs in the SF might be caused by the long-term joint dislocation, leading to a pro-chondrogenic microenvironment for the SMSC in the SF and further causing chondrogenic gene changes in the cartilage and other joint tissues. Further studies on the comprehensive miRNA expression profiles would help elicit how miRNAs and downstream chondrogenic genes were dysregulated in DDH development. There are some limitations to our study. GAPDH was used as a reference gene in our study as previously reported under the assumption that GAPDH expression was stable across tissue conditions [17, 38], but recent works have demonstrated that the expression of GAPDH is actually inconsistent [39]. When choosing a reference gene, it would be better to use other stable genes like PPIA or HPRT1 to crosscheck the expression of GAPDH and further validate the expression of the chondrogenesis-related genes mentioned in our study [40, 41]. Second, the two loci in our study were in the intron region of the FRZB gene. In this case, no association study was performed between loci genotype and the corresponding expression of FRZB. Further exploration of functional mutations in the promoter or exon regions would help uncover rare FRZB mutations responsible for abnormal FRZB expression in DDH patients.

## Supplementary Material

Supplementary Table 1
