# Supplementary material for: Secreted frizzled-related protein 3 was genetically and functionally associated with developmental dysplasia of the hip
Source: Aging (Albany NY). 2021 Apr 4;13(8):11281–95. doi: 10.18632/aging.202815 (PMC8109121; doi:10.18632/aging.202815)
Supplement: Supplementary Table 1 [file aging-13-202815-s001.pdf]

SUPPLEMENTARY TABLE

Supplementary Table 1. Primer sequences for genes in PCR assay.

	Gene	Forward primers (from 5' to 3')	Reverse primers (from 5' to 3')
1	SOX9	CCACGGAACAGACTCACA	GAGATTGCCCAGAGTGCT
2	COL1A1	GCTCCCTTGGACATTGGT	GGAAAAGTGGGCTGGGT
3	COL2A1	TGGCGAGAAAGGAGAACC	CAGGCAGACCAACAATGC
4	β-catenin	GAAGGCGTGGCAACATAC	GTCCTGAAGAGGGAACTGG
5	WNT3A	GCAGAGCCAGGGTTGTTT	AGAGAAGCTCGGGACAGG
6	WNT8A	CCTGATAACCCGTCCCAA	GACAATTCCACCGTTCCC
7	MMP9	CACCGGCTAAACCACCTC	CGCCCGACACACAGTAAG
8	MMP13	GGGGAGCCACAGATGAG	AACGCTCGCAGTGAAAAG
9	ADAMTS5	TGGTGAAGGTGGTGGTG	GCTGGTTATGTTGGTGCTG
10	COL10A1	ATAGGCAGCAGCATTACGA	CCCAAACGTGAGTCCCT
11	RUNX2	GCCGGGAATGATGAGAAC	TGGGGAGGATTTGTGAAGA
12	OCN	CAGTCCCCAGCCCAGAT	GCGTTTGTAGGCGGTCTT
13	FRZB	TCTGGCTTCCTCTTCTGC	GCTCTTCCCAAGCGTCTA
14	ACAN	CCTATGAGGCAGGCTATGA	CTGGGCTGCTGTCTTTG
15	ITGA4	CTGGCTCTCTAATGCCTCA	TTCCCACAAGGCTCTCC
16	ASPN	GCAAAAGTGGGAGTGAATG	GCCAAGAACACAACGAAAT
17	COL3A1	AAAGAATGGGGAGACTGGA	TGCCTTGTAATCCTTGTGG
18	SFRP4	CACCTATCCCTCGAACACA	CATCCTTGAACGCCACTC
19	THBS2	TCGTTGGAGACCAGTGTG	TCAGAGTCGCAGGCATC
20	ITGB1	CTTATTGGCCTTGCCTTG	CCCACTTGGCATTCATTT
21	ITGA1	TGTGTGTGATGCCCAGAG	TCCCAGAAGCCCTAGCA
22	ITGAV	AACCAATTAGCAACACGGA	ATCTTCAATGCCGTCACC
23	ITGA8	CGGGGCACCTCTCTTTA	ATCTCCCAAATGTCTCCGT
24	PTK2	CGCTTTCTCCCTTTGGT	CCCTGGCTTCATCTATTCC
25	miR-454	ACCCTATCAATATTGTCTCTGC	TGGTGTCGTGGAGTCG
26	miR-130a	CCAGGGCTTTTCAAAAATGA	CCGATCCAATCTGTTCTGGT
27	miR-130b	TTCACATTGTGCTACTGTCTGC	GCTCTGACTTTATTGCACTACT
28	miR301a	ACACTCCAGCTGGGGCTCTGACTTTATTGCAC	CTCAACTGGTGTCGTGGA
29	miR-301b	ATACTCGAGATCCTAGTTTGATACTCCCAGTCTT	TGTTCTAGACATATTTACTTTTATATTTCCATAC
30	U6	CTCGCTTCGGCAGCACA	AACGCTTCACGAATTTGCGT
